# Ayurveda-based Botanicals as Therapeutic Adjuvants in Paclitaxel-induced Myelosuppression

**DOI:** 10.3389/fphar.2022.835616

**Published:** 2022-02-22

**Authors:** Akash Saggam, Prathamesh Kale, Sushant Shengule, Dada Patil, Manish Gautam, Girish Tillu, Kalpana Joshi, Sunil Gairola, Bhushan Patwardhan

**Affiliations:** ^1^ AYUSH-Center of Excellence, Center for Complementary and Integrative Health, School of Health Sciences, Savitribai Phule Pune University, Pune, India; ^2^ Serum Institute of India Pvt. Ltd., Pune, India; ^3^ Department of Biotechnology, Sinhgad College of Engineering, Pune, India

**Keywords:** Ayurveda, *Asparagus racemosus*, *Withania somnifera*, Cancer Therapeutics, Chemotherapy, Paclitaxel, Myelosuppression, Cytokine Modulation

## Abstract

Chemotherapy-induced myelosuppression is one of the major challenges in cancer treatment. Ayurveda-based immunomodulatory botanicals *Asparagus racemosus* Willd (AR/Shatavari) and *Withania somnifera* (L.). Dunal (WS/Ashwagandha) have potential role to manage myelosuppression. We have developed a method to study the effects of AR and WS as therapeutic adjuvants to counter paclitaxel (PTX)-induced myelosuppression. Sixty female BALB/c mice were divided into six groups—vehicle control (VC), PTX alone, PTX with aqueous and hydroalcoholic extracts of AR (ARA, ARH) and WS (WSA, WSH). The myelosuppression was induced in mice by intraperitoneal administration of PTX at 25 mg/kg dose for three consecutive days. The extracts were orally administered with a dose of 100 mg/kg for 15 days prior to the induction with PTX administration. The mice were observed daily for morbidity parameters and were bled from retro-orbital plexus after 2 days of PTX dosing. The morbidity parameters simulate clinical adverse effects of PTX that include activity (extreme tiredness due to fatigue), behavior (numbness and weakness due to peripheral neuropathy), body posture (pain in muscles and joints), fur aspect and huddling (hair loss). The collected samples were used for blood cell count analysis and cytokine profiling using Bio-Plex assay. The PTX alone group showed a reduction in total leukocyte and neutrophil counts (4,800 ± 606; 893 ± 82) when compared with a VC group (9,183 ± 1,043; 1,612 ± 100) respectively. Pre-administration of ARA, ARH, WSA, and WSH extracts normalized leukocyte counts (10,000 ± 707; 9,166 ± 1,076; 10,333 ± 1,189; 9,066 ± 697) and neutrophil counts (1,482 ± 61; 1,251 ± 71; 1,467 ± 121; 1,219 ± 134) respectively. Additionally, higher morbidity score in PTX group (7.4 ± 0.7) was significantly restricted by ARA (4.8 ± 1.1), ARH (5.1 ± 0.6), WSA (4.5 ± 0.7), and WSH (5 ± 0.8). (Data represented in mean ± SD). The extracts also significantly modulated 20 cytokines to evade PTX-induced leukopenia, neutropenia, and morbidity. The AR and WS extracts significantly prevented PTX-induced myelosuppression (*p* < 0.0001) and morbidity signs (*p* < 0.05) by modulating associated cytokines. The results indicate AR and WS as therapeutic adjuvants in cancer management.

## Introduction

Cancer is one of the leading causes of mortality worldwide. The GLOBOCAN 2020 data indicates more than 19 million new cases with more than 9 million deaths due to cancer in the year 2020 with increasing prevalence globally (GLOBOCAN 2020 Fact Sheet 2020). The therapeutic approaches of cancer include chemotherapy, radiotherapy, and surgery depending upon the stage of cancer and response to therapies. In recent years, immunotherapy has emerged as an effective therapeutic alternative (Arruebo et al., 2011). However, chemotherapy is a predominant approach in cancer therapeutics that restricts cancer development by using chemotherapeutic agents. The chemotherapeutic agents include alkylating agents, cytoskeletal disrupters, anthracyclines, and enzyme inhibitors etc. (Shewach and Kuchta 2009). Although chemotherapy has emerged as an effective therapeutic approach, there are several dose-limiting adverse effects (Chopra et al., 2016; Regnard and Kindlen 2019). Chemotherapy-induced myelosuppression is one of the major challenges in cancer therapeutics. The declined ability of bone marrow to produce blood cells is referred as myelosuppression that leads to neutropenia, leukopenia, and thrombocytopenia (Maxwell and Maher 1992).

In the present study, we used paclitaxel (PTX/taxane) as a prototype chemotherapeutic drug to induce myelosuppression in a mouse model. PTX is one of the extensively used anticancer drugs in the treatment of various types of cancers including breast cancer, ovarian cancer, pancreatic cancer, advanced non-small cell lung cancer, and liver cancer (Weaver 2014). The pharmacological activity of PTX is to stabilize the microtubules of cancer cells that leads to tumor suppression; however, the adverse effects of PTX remains a major concern. The clinical adverse effects of PTX include myelosuppression, fatigue, hypersensitivity reactions, hair loss, peripheral neuropathy (numbness and weakness), pain in muscles and joints etc. (Markman 2003). PTX is known to inhibit the proliferation of bone marrow cells. It induces apoptosis by activating caspase-3/7 and promotes DNA damage leading to myelosuppression (Hu et al., 2016). The hematopoietic growth factors especially granulocyte colony-stimulating factor (G-CSF) and granulocyte-macrophage colony-stimulating factor (GM-CSF) are employed in between chemotherapeutic cycles that increase blood cell count in patients to manage myelosuppression (Dale 2002). However, the notable toxicity risks limit the use of growth factors (Puhalla, Bhattacharya, and Davidson 2012). Additionally, active involvement of growth factors in carcinogenesis is speculated by researchers (Halper 2010). In such a scenario, a safe therapeutic adjuvant with better effectiveness to counter chemotherapy-induced myelosuppression is needed.

Ayurveda, a traditional medicine system, has a lot to offer to tackle myelosuppressive indications. The therapeutic approaches of Ayurveda focus on balancing the physiological processes and body constitution leading to homeostasis (Patwardhan et al., 2015). Ayurveda medicines have a long history of safe use and are being used in cancer management (Lele 2010; Raut et al., 2012; Patwardhan 2014). The *Rasayana* approach of Ayurveda strengthens physiological homeostasis through rejuvenating and adaptogenic effects (Chulet and Pradhan 2009). Ayurveda botanicals, *Asparagus racemosus* Willd. (AR) known as Shatavari and *Withania somnifera* (L.). Dunal (WS) known as Ashwagandha are *Rasayana* botanicals used as adaptogens and immunomodulators (Balasubramani et al., 2011). AR and WS are known for their multimodal pharmacological activities such as galactagogic, anti-ageing, nootropic, anti-inflammatory, and especially immunomodulation (N. Singh et al., 2011; Alok et al., 2013). The immunomodulatory activity of AR and WS can help to counter chemotherapy-induced myelosuppression. AR and WS have been reported to offer myeloprotection during cancer chemotherapy (Diwanay et al., 2004). The experimental methods replicated *Rasayana* approach of Ayurveda that recommends pre-administration and daily dosing of botanical extracts (Chulet and Pradhan 2009).

The present study reports a method and effects of AR and WS as therapeutic adjuvants to counter PTX-induced myelosuppression in BALB/c mice. This study explores the preventive effects of AR and WS extracts in PTX-induced myelosuppression. It also investigates the observational parameters of morbidity that simulate the clinical adverse effects of PTX as mentioned above. Furthermore, it also connects the myelosuppression and morbidity parameters to cytokine modulation.

## Materials and Methods

### Materials

The commercially available clinical formulation of PTX- Mitotax®-300 (Dr Reddy’s Laboratory) was procured from local pharmacists and was standardized for dosage regimen. The Cremophor EL^®^ (polyoxyethylated castor oil) (Cat. No. 238470) and absolute ethanol were obtained from Millipore and SRL respectively. The standardized aqueous and hydroalcoholic extracts of *Asparagus racemosus* (ARA, ARH) and *Withania somnifera* (WSA, WSH) were obtained from Pharmanza Herbal Pvt. Ltd. as a gift. We have used standardized extracts prepared as per previously reported method (Borse et al., 2021).

### Animals

The ethics approval (27/23-03/2021-R, dated-23/03/2021) was obtained from the Institutional Animal Ethics Committee of Serum Institute of India Pvt. Ltd. The female BALB/c mice were reared as per CPCSEA guidelines in the animal house of Serum Institute of India Pvt. Ltd. with *ad libitum* food and water. The mice weighing 13-18 gms were housed in polypropylene cages on ground corn cobs bedding at optimum room temperature with 12 h of light/dark cycle. The mortality and morbidity variables in the method development experiment suggest that the female mice were more tolerant to the high and consecutive doses of PTX. The model titers the dose regimen to balance myelosuppression and survival. Therefore, we used female mice in the present study.

### Dosing

**FIGURE 1:**
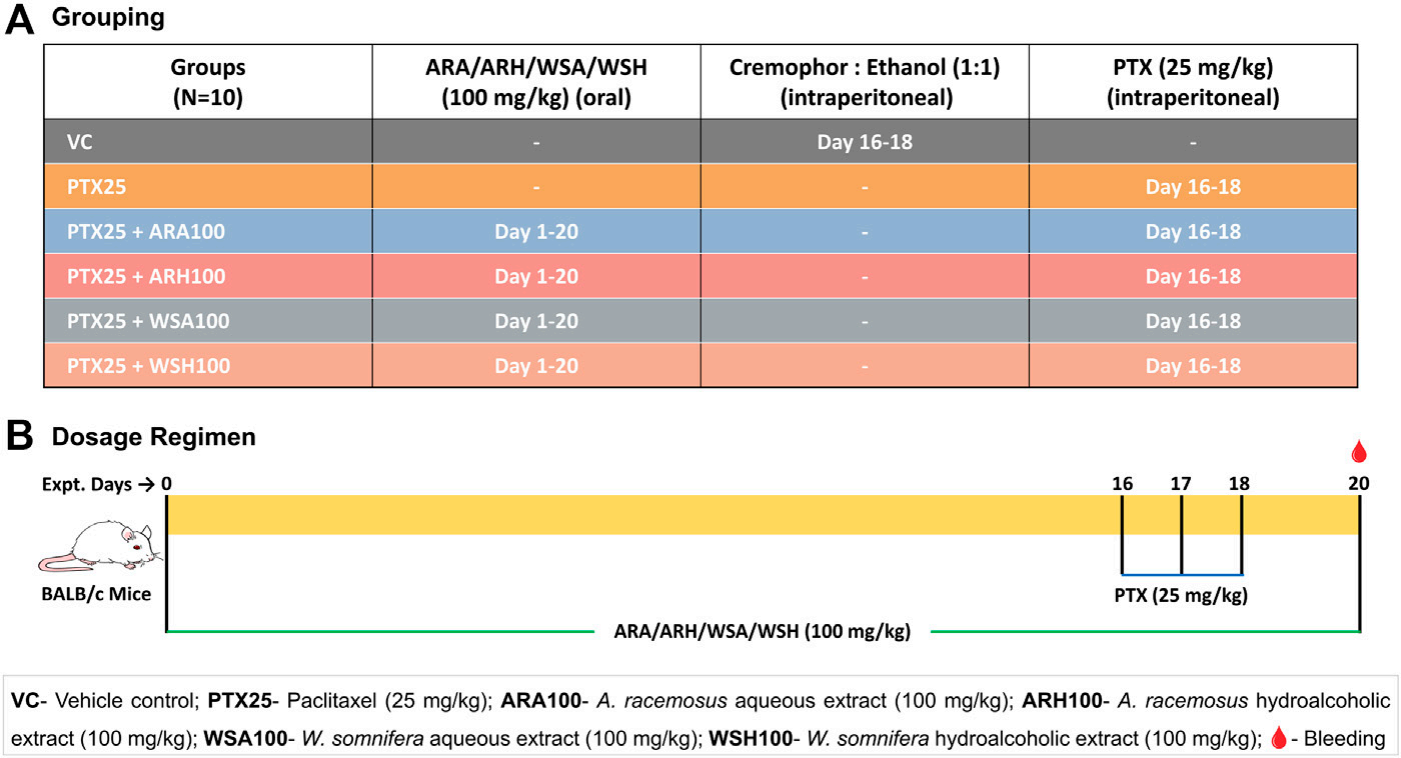
Grouping and dosage regimen. **(A)** depicts grouping of mice to be dosed with botanical extracts and PTX for desired days. **(B)** illustrates the dosing regimen of botanical extracts as per Ayurveda (for 20 days) and that of PTX (on 16th, 17th and 18th day) as standardized for induction of myelosuppression. The blood samples were collected from retro-orbital plexus on 20th day for cell count and cytokine analysis.

The animals were randomly allocated in 6 groups-vehicle control (VC), PTX alone (PTX), and PTX along with aqueous and hydroalcoholic extracts of AR (PTX + ARA and PTX + ARH) and WS (PTX + WSA and PTX + WSH) (Figure 1A). Each group contained 10 mice weighing 13-18 gms.

The 59.125 mg/kg PTX dose per mouse is equivalent to that of the human dose of 175 mg/m^2^. Although PTX is administered intravenously in humans, it was given through the intraperitoneal route in mice. Our earlier standardization experiments have suggested consistent results with intraperitoneal dosing in small animal models like mice. It also has a practical advantage over intravenous administration in mice (Ray et al., 2011). The commercially available clinical formulation of PTX was diluted with sterile saline to obtain the desired concentration. The mouse dose of botanical extracts was extrapolated from recommended clinical dose based on Ayurveda (Gogte 2000). Moreover, previous studies by the investigators’ group have consistently demonstrated that the dose of 100 mg/kg of AR and WS extracts for consecutive 15 days is best for immunomodulation (Ziauddin et al., 1996; Gautam, et al., 2004a; Gautam, et al., 2004b).

As shown in the dosage regimen (Figure 1B), the mice were pre-administered with 100 mg/kg of botanical extracts orally every morning for 15 days prior to PTX dosing and continued throughout the experiment (up to 20th day). After a series of dose standardization experiments, 25 mg/kg of PTX dose was finalized to be administered intraperitoneally for 3 consecutive days (15th, 16th, and 17th day) to induce myelosuppression. The VC group was dosed with 1:1 solution of cremophor and absolute ethanol in the dose equivalent to the PTX since the commercially available PTX was dissolved in cremophor-ethanol solution. The mice were anesthetized by drop jar method using isoflurane and were bled from retro-orbital plexus 2 days after PTX cycle (on 20th day) (Anesthesia Guidelines Mice, 2021). These samples were analysed for blood cell count and plasma cytokine profiling.

### Blood Cell Count Analysis

The primary outcome measures of myelosuppression were total leukocyte and neutrophil counts as indicators of myelosuppression. The blood collected in EDTA-coated vacutainers was subjected to Sysmex XN550 analyser. The total leukocyte count/mm^3^ (TLC) and absolute neutrophil count/mm^3^ (ANC) were considered for further analysis.

### Morbidity Analysis

Along with the total leukocyte and neutrophil count, we also considered examining other clinical adverse effects of PTX in mice. Cancer chemotherapy especially with PTX is associated with several adverse effects (Marupudi et al., 2007). Such adverse effects may be the signs of morbidities. Previous studies have reported the methods for observational scoring in cancer therapeutics (Banipal et al., 2017; Banerjee and Prasad 2020). Therefore, the secondary outcome measures included morbidity parameters such as activity, behavior, fur aspect, huddling, and posture. The morbidity parameters simulated clinical adverse effects of PTX such as fatigue (extreme tiredness), peripheral neuropathy (numbness and weakness), hair loss (alopecia), and pain in muscles and joints (Batchelder et al., 1983; Paclitaxel Taxol, 2020; Clinical Signs of Pain and Disease in Laboratory Animals 2021) (Table 1).

**TABLE 1:** Rationale of morbidity analysis.

Clinical adverse effects of PTX	Observations and signs in animals	Parameters
Fatigue (extreme tiredness)	Fatigue reduces general wandering of animal in cage	Activity
Peripheral neuropathy (numbness and weakness)	Abnormal behavior and lack of relocation is due to numbness	Behavior
Hair loss (alopecia)	Ruffled and fur loss represents alopecia	Fur aspect
	Adult mice huddle in response to cold caused by hair loss	Huddling/Grouping
Painful muscles and joints	Hunched posture is a general sign of pain or distress	Posture

References: (Batchelder et al., 1983; Paclitaxel Taxol, 2020; Clinical Signs of Pain and Disease in Laboratory Animals 2021).

The mice from all the groups were observed daily for morbidity parameters including activity, behavior, fur aspect, huddling, and body posture throughout the experiment. The morbidity scoring (Table 2) was derived based on literature survey (Banipal et al., 2017; Banerjee and Prasad 2020; Paclitaxel Taxol, 2020; Clinical Signs of Pain and Disease in Laboratory Animals, 2021; Batchelder et al., 1983) wherein score ‘0’ represents normal and score ‘3’ represents highest observed morbidity parameter. More the score indicates higher morbidity. The morbidity score of each group was calculated as the average of the sum of all the parameters of each mouse of the group (data are shared as Supplementary Material S1).

**TABLE 2:** Morbidity scoring system.

Score	0	1	2	3
Activity	Normal	Reduced	Only when provoked	Little or none with provocation
Behavior	Normal	Slow normal when disturbed	Abnormal when disturbed and relocation	Abnormal when disturbed and no relocation
Fur aspect	Normal coat/actively grooming	Slightly ruffled or mild loss of fur	Ruffled fur and moderate loss of fur	Ruffled fur, piloerection, and significant loss of fur
Huddling/Grouping	Normal or no grouping	Mild grouping or social proximity	Moderate grouping or social proximity	High grouping or social proximity
Posture	Normal	Slightly hunched but moving freely	Hunched with stiff movement	Hunched with little or no movement

References: (Banipal et al., 2017; Banerjee and Prasad 2020; Paclitaxel Taxol, 2020; Clinical Signs of Pain and Disease in Laboratory Animals 2021; Batchelder et al., 1983).

### Cytokine Profiling

The panel of 20 cytokines (eotaxin, G-CSF, GM-CSF, IFN-γ, IL-1α, IL-1β, IL-2, IL-3, IL-4, IL-5, IL-6, IL-10, IL-13, IL-17A, KC, MCP-1, MIP-1α, MIP-1β, RANTES, and TNF-α) was considered for cytokine profiling. Cytokines were measured using Bio-Plex multiplex that operates on the principle of capture sandwich immunoassay. The polystyrene magnetic beads coupled with antibodies of specific cytokines were incubated with the plasma samples on shaker at 850 ± 50 rpm for 30 min in a 96-well plate. The beads were washed thrice using wash buffer. The primary antibody-cytokine conjugate was incubated with secondary (detection) antibody for 30 min followed by washing thrice using wash buffer. Further, the secondary antibodies were tagged with streptavidin-phycoerythrin and incubated for 10 min. The beads were again washed thrice and resuspended in buffer. The plate was then subjected to Bio-Plex 200 system for reading.

### Statistical Analysis

The experimental results of blood cell count, morbidity analysis, and cytokine profiling were analysed using GraphPad 9.0.0 software. The statistical significance of the data of blood cell count analysis (TLC and ANC), morbidity score, and observed concentrations of cytokines (pg/ml) was analysed by one-way ANOVA. The intergroup comparison was carried out using Tukey’s statistical hypothesis testing with 95% confidence interval. The data is represented in mean ± SD.

## Results

The mice were observed healthy with normal activity and behavior at the time of housing.

### AR and WS Prevented PTX-induced Myelosuppression

**FIGURE 2:**
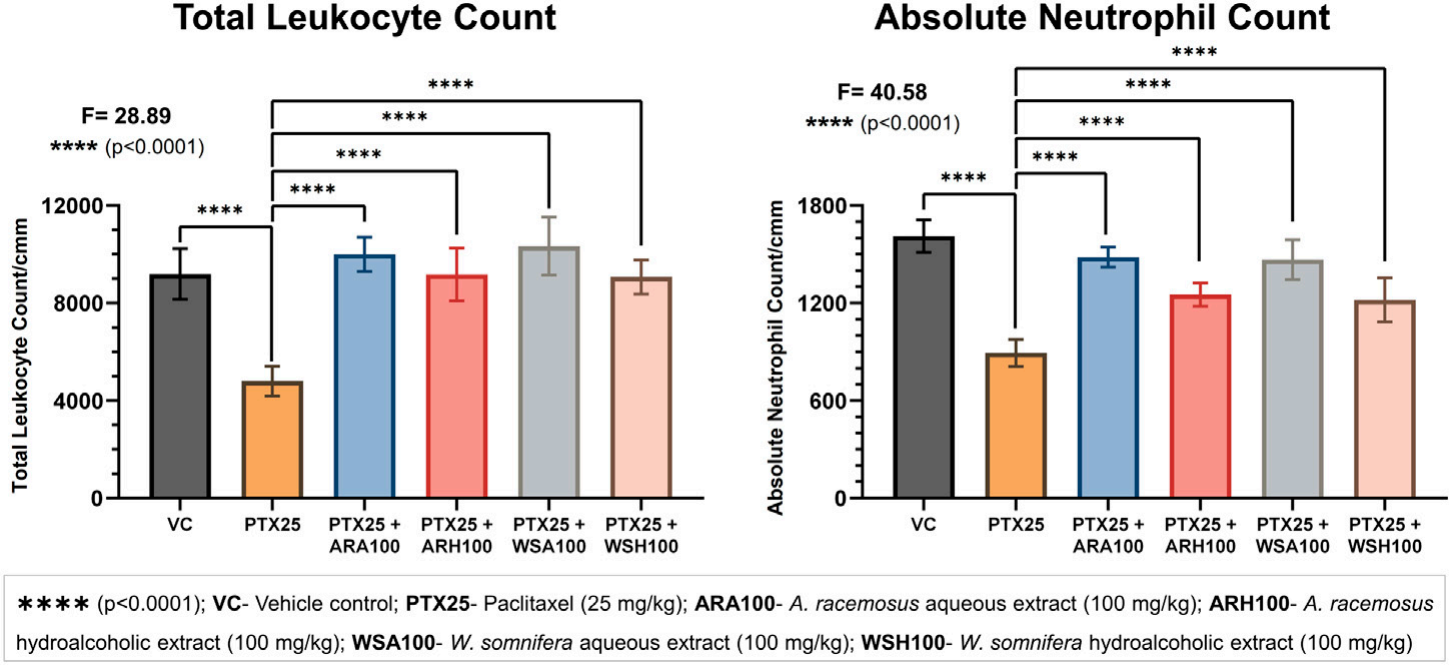
Blood cell count analysis. The figure depicts that ARA, ARH, WSA, and WSH significantly prevented PTX-induced leukopenia and neutropenia. PTX at 25 mg/kg dose significantly (*p* < 0.0001) reduces the total leukocyte and neutrophil count. Pre-administration of botanical extracts ARA, ARH, WSA, and WSH at 100 mg/kg dose 15 days prior to PTX dosing significantly (*p* < 0.0001) prevents the reduction in total leukocyte and neutrophil count. All botanical extracts normalize the total leukocyte and neutrophil count comparable to VC group. The data points are represented as mean ± SD with n = 6.

The pre-administration of botanical extracts significantly (p < 0.0001) prevented PTX-induced myelosuppression in BALB/c mice (Figure 2). The PTX group showed a reduction in total leukocyte and neutrophil counts (4,800 ± 606; 893 ± 82) when compared with that of a VC group (9,183 ± 1,043; 1,612 ± 100) respectively. The PTX-induced leukopenia and neutropenia were evident by reduced counts of total leukocytes and neutrophils respectively. The adjuvant groups with botanical extracts were found to maintain these counts. Pre-administration of ARA, ARH, WSA, and WSH extracts prevented PTX-induced reduction by normalizing total leukocyte counts (10,000 ± 707; 9,166 ± 1,076; 10,333 ± 1,189; 9,066 ± 697) and neutrophil counts (1,482 ± 61; 1,251 ± 71; 1,467 ± 121; 1,219 ± 134) respectively. The total leukocyte and neutrophil counts of adjuvant groups were adjacent to that of VC group. Thus, ARA, ARH, WSA, and WSH prevented leukopenia and neutropenia induced by PTX in BALB/c mice.

### AR and WS Reduced PTX-induced Morbidity

**FIGURE 3:**
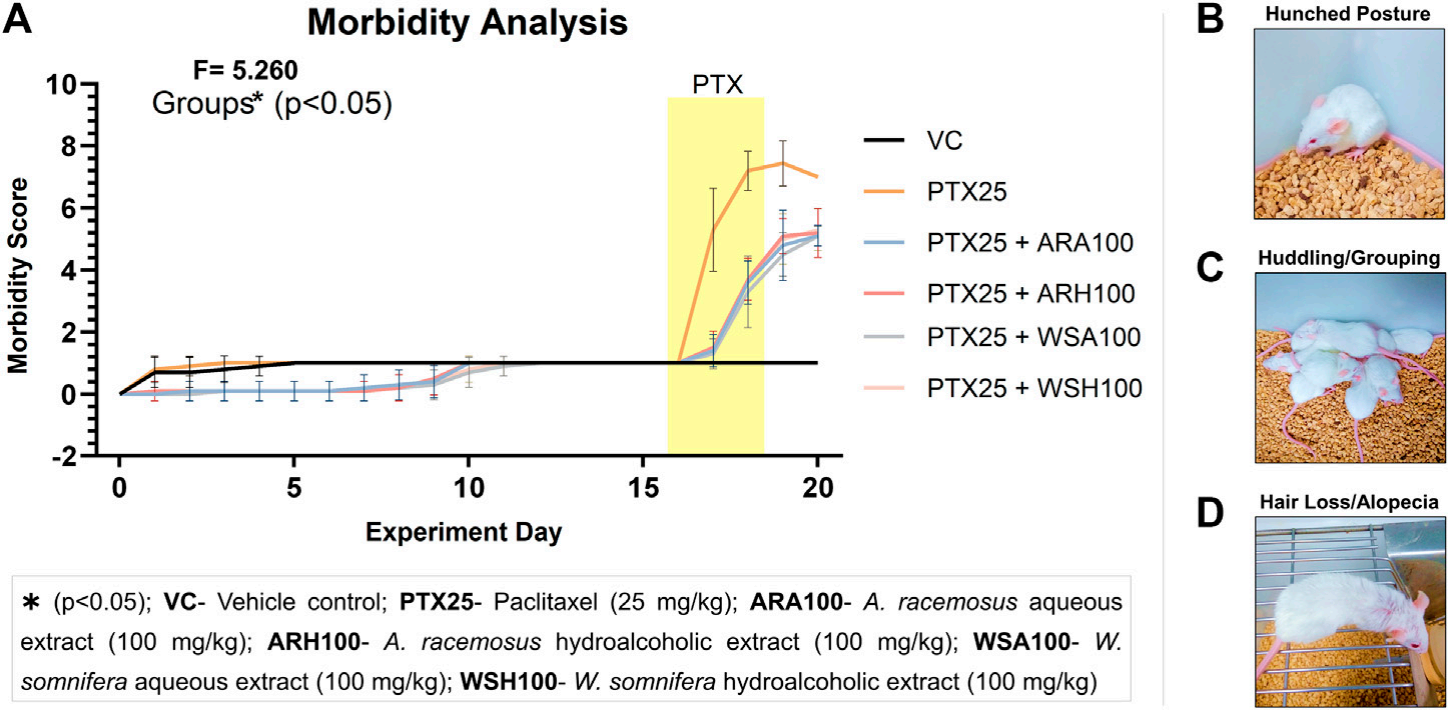
Morbidity analysis. The figure represents that ARA, ARH, WSA, and WSH significantly prevented PTX-induced morbidity. **(A)** shows the graphical representation of morbidity score based on observations of mice. These observations included reduced activity and relocation, hunched posture **(B)**, huddling/grouping **(C)**, hair loss/alopecia **(D)** that simulated clinical adverse effects of PTX. The morbidity score of each group was calculated as the average of the sum of all the parameters of each mouse of the group. Therefore, more the score more would be the morbidity signs in mice. The yellow patch depicts the duration of PTX dosing on 16th, 17th, and 18th day of the experiment. The graph shows that consecutive dosing of PTX (25 mg/kg) increases morbidity score in mice from 16th day up to 20th day of the experiment. Pre-administration of botanical extracts ARA, ARH, WSA, and WSH at 100 mg/kg dose 15 days prior to PTX dosing significantly (*p* < 0.05) restricts the increased morbidity score. The data points are represented as mean ± SD with n = 10.

The pre-administration of botanical extracts significantly (*p* < 0.05) reduced PTX-induced morbidity signs in BALB/c mice (Figure 3A). These signs included reduced activity and relocation, hunched posture (Figure 3B), huddling/grouping (Figure 3C), hair loss/alopecia (Figure 3D) that simulated clinical adverse effects of PTX. The morbidity score was significantly higher in PTX group (7.4 ± 0.7) than that of VC group (1 ± 0). The clinical adverse effects of PTX were observed in mice such as reduced activity or general wandering (fatigue), abnormal behavior and lack of relocation (numbness and weakness due to peripheral neuropathy), hunched posture (pain in muscles and joints), notable hair loss and grouping due to cold (alopecia). The adjuvant groups with botanical extracts restricted the aforementioned morbidity signs. ARA (4.8 ± 1.1), ARH (5.1 ± 0.6), WSA (4.5 ± 0.7), and WSH (5 ± 0.8) decreased the morbidity score that was elevated by PTX. In all the adjuvant groups (those treated with extracts), it was observed that the activity and relocation of mice was improved with reduced stiffness of posture followed by negligible hair loss and grouping.

### AR and WS Modulated PTX-induced Cytokine Alterations

**FIGURE 4:**
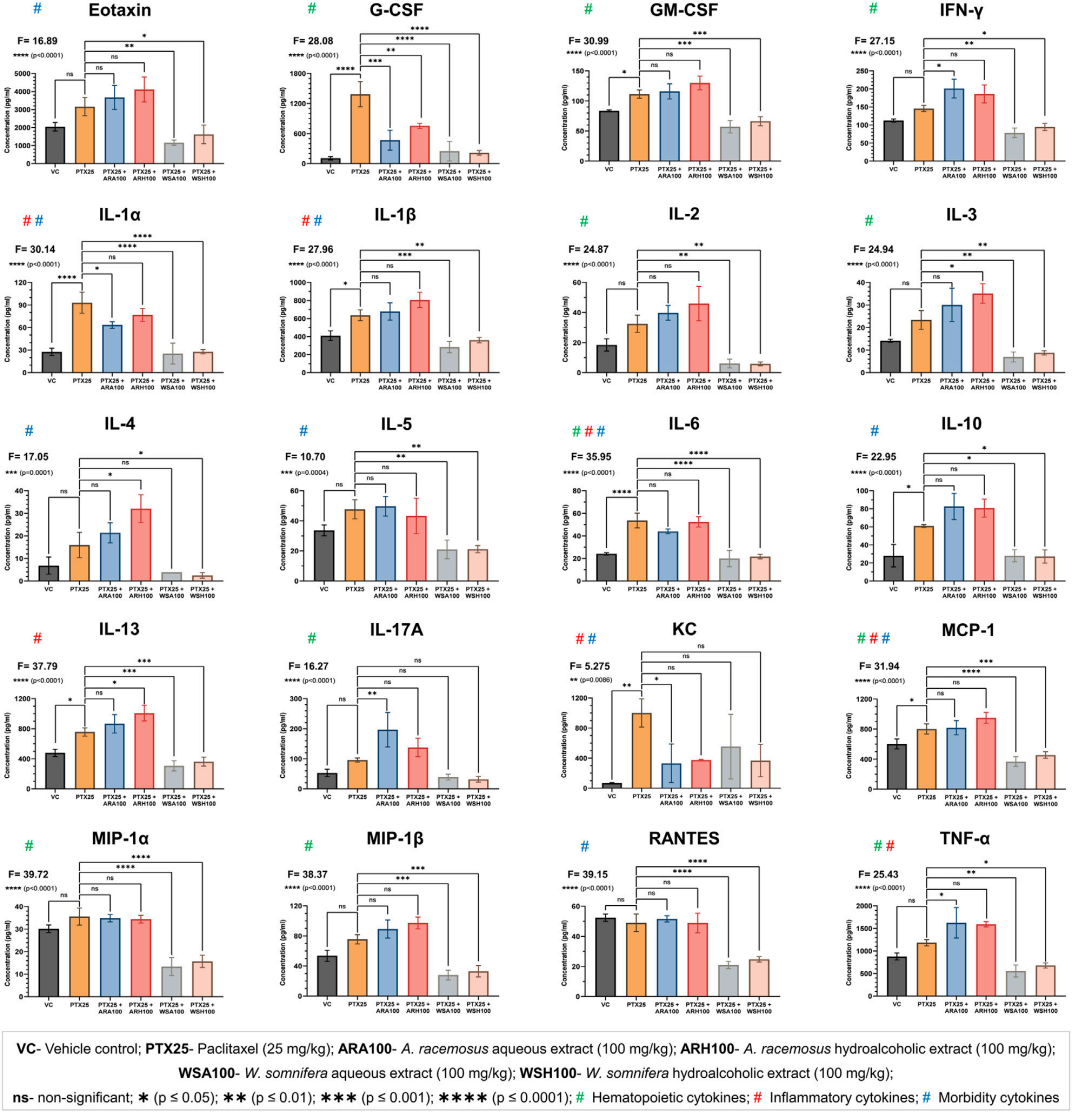
Cytokine profiling. The figure shows that ARA, ARH, WSA, and WSH extracts modulated the plasma levels of cytokines altered by PTX-administration with different statistical significance (**p = 0.0086 to ****p < 0.0001). The cytokines are categorized by their involvement in the biological processes such as hematopoiesis, inflammation, and morbidity signs (fatigue, peripheral neuropathy, hair loss, and pain in muscles and joints). PTX administration (25 mg/kg) for three consecutive days significantly altered the plasma levels of cytokines. Pre-administration of ARA (G-CSF, IL-1α, and KC) and ARH (G-CSF) extracts (100 mg/kg) reversed the expressions of cytokines induced by PTX. Pre-administration of WSA and WSH extracts (100 mg/kg) normalized the PTX-altered cytokine levels comparable to that of VC group. The data points are represented as mean ± SD with n = 6.

The pre-administration of botanical extracts significantly (p=0.0086 for KC, p=0.0004 for IL-5, p=0.0001 for IL-4, and p<0.0001 for all other cytokines) mitigated PTX-induced cytokine alterations in BALB/c mice (Figure 4). PTX significantly (p<0.05) increased the plasma levels of cytokines (G-CSF, GM-CSF, IL-1α, IL-1β, IL-6, IL-10, and IL-13) and chemokines (KC and MCP-1). The botanical extracts modulated the PTX-induced cytokine alterations to maintain homeostasis. ARA and/or ARH reduced the expression of G-CSF, IL-1α and increased the expression of IL-13. WSA and WSH also reduced the expression of G-CSF, GM-CSF, IL-1α, IL-1β, IL-6, IL-10, and IL-13. Overall, WS was found to normalise the PTX-induced cytokine alterations to bring it back to the level of VC. ARA was found to decrease KC and increase TNF-α and IL-17A whereas ARH increased IL-3 and IL-4 levels in mouse plasma significantly (p<0.05).

## Discussion

The present study reports a method developed for induction of myelosuppression using chemotherapeutic drug PTX. The standardized PTX dose of 25 mg/kg (administered intraperitoneally for three consecutive days) significantly reduces total leukocyte and neutrophil count in mice. An application of clinically used PTX formulation mimics the model closer to the clinical scenarios. Therefore, this mouse model may be a better evidence-based option over reported predictive models of myelosuppression (Schurig et al., 1985; Schurig, Florczyk, and Bradner 1986; Friberg et al., 2002; Friberg and Karlsson 2003). The methods of this study are useful in screening therapeutic adjuvants in cancer chemotherapy. The data from this study suggests that AR and WS extracts prevented PTX-induced myelosuppression. The ARA, ARH, WSA, and WSH extracts significantly evaded the leukopenia, neutropenia, and morbidity induced by PTX in BALB/c mice. This preventive effect is corroborated with cytokine data (Figure 4).

### AR and WS Restrict PTX-induced Inflammatory Response

PTX was found to induce inflammatory conditions evident by significant expression of pro-inflammatory cytokines (IL-1α, IL-1β, IL-6, and IL-13) and chemokines (KC, and MCP-1). AR and WS extracts and their phytoconstituents have been reported as potential anti-inflammatory agents acting through inhibition of pro-inflammatory cytokines released by several immune cells including monocytes, macrophages, dendritic cells, and keratinocytes etc. (Agarwal et al., 1999; Bani et al., 2006; Alok et al., 2013; Tiwari et al., 2017; Dubey et al., 2018; Saggam et al., 2021). In this study, the PTX-induced inflammation was significantly prevented by AR (downregulation of IL-1α and KC) and WS (downregulation of IL-1α, IL-1β, IL-6, IL-13, and MCP-1).

### AR and WS Maintain Hematological Homeostasis

The AR and WS extracts prevented PTX-induced decrease in total leukocyte and neutrophil count in mice through mitigation of cytokine alterations. The increased plasma level of G-CSF is well correlated with cyclic neutropenia clinically. The neutrophil count was found to be inversely proportional to the plasma G-CSF levels (Watari et al., 1989). The present study also demonstrated the PTX-induced neutropenia accompanied by the increased plasma level of G-CSF. Both of these parameters were found to reverse in all the adjuvant groups with AR and WS extracts. Furthermore, a clinical study reported an increase in IL-6, GM-CSF, IFN-γ levels in breast cancer patients undergoing PTX treatment (Tsavaris et al., 2002). The results of this study also support the significant increase in IL-6 and GM-CSF on PTX administration that was found to be normalised by WS. However, the increase in IFN-γ remained non-significant in PTX alone group.

TNF-α plays a central role in physiological homeostasis by regulating immunity (Silke and Hartland 2013). During an inflammatory condition, TNF-α maintains the persistence of hematopoietic stem cells and ensures myeloid regeneration (Yamashita and Passegué 2019). PTX administration triggered TNF-α expression that was found to be enhanced by ARA extract to maintain regeneration of blood cells matured from myeloid lineage. ARA extract also synergistically increased the expression of IL-17A that contributes to granulopoiesis and stimulates mobilization of mature granulocytes into the circulation (Krstic et al., 2012; Mojsilović et al., 2015). ARH extract facilitated the expression of IL-2 and IL-3 that promotes T-cell proliferation and granulocyte colony formation respectively (Groopman, Molina, and Scadden 1989; Murphy 1993; Metcalf 2008).

The results of present study suggested a significant increase in plasma levels of MCP-1 induced by PTX. The MCP-1 is reported for regulating monocyte infiltration from peripheral blood to surrounding tissue through vascular endothelium as an inflammatory response (Deshmane et al., 2009). Moreover, PTX is also known to trigger inflammation mediated through pro-inflammatory cytokines (Ilinskaya, Dobrovolskaia, and McNeil 2012). This underlines the possibility of PTX to induce monocyte depletion in peripheral blood as a result of inflammatory response through MCP-1. WS on the other hand reduces PTX-induced increase in MCP-1 levels and may contribute to maintaining monocyte homeostasis in peripheral blood. Additionally, WS reduced the expression of MIP-1α that inhibits the proliferation of hematopoietic stem cells (Cook 1996) and AR upregulated MIP-1β that reverses the myelosuppressive effect of MIP-1α (Broxmeyer et al., 1993). These results indicate the corrective measures of AR and WS in PTX-induced myelosuppression.

PTX induces IL-1β overexpression that subsequently promotes the invasiveness of malignant cells. Blocking IL-1β pathway using IL-1 receptor antagonist followed by PTX therapy slightly inhibits tumor growth (Voloshin et al., 2015). Therefore, an adjuvant that inhibits IL-1 cytokines along with PTX would be beneficial. The results of this study showed a significant reduction of IL-1α and IL-1β by AR and WS extracts induced by PTX. This indicates adjuvant potential of AR and WS in cancer therapeutics.

### AR and WS Ameliorate PTX-induced Morbidity

The morbidity implications of PTX such as fatigue, peripheral neuropathy, hair loss, pain in muscles and joints were also restricted by AR and WS. Clinically, the increase in IL-6 and IL-10 on PTX administration is correlated with joint pain and fatigue respectively in cancer patients (Pusztai et al., 2004). Our study showed the increased morbidity score for hunched posture and reduced activity due to joint pain and fatigue respectively in mice. It was also confirmed by the increased levels of IL-6 and IL-10 in mouse plasma after PTX administration. WS significantly reduced the observed morbidity and plasma levels of IL-6 and IL-10 in mice.

Cytokine-induced inflammation is an established physiological link between fatigue and pain (Louati and Berenbaum 2015; Karshikoff, Sundelin, and Lasselin 2017). On the other hand, cancer and chemotherapy-associated persistent fatigue is correlated with the increased levels of IL-1 and IL-6 (Bower 2014, 2007). Pain in muscles and joints is also associated with pro-inflammatory cytokines viz. IL-1 and IL-6 that acts either through sensory neurons or prostaglandin mediators (Ren and Torres 2009; Schaible 2014). The AR and WS extracts have ameliorated PTX-induced signs of fatigue (decreased wandering of mice in cages) and pain (hunched posture) that is evident by decreased plasma levels of IL-1 and IL-6. The patients with joint pain due to musculoskeletal disorder found to have greater levels of eotaxin (J. Singh, Noorbaloochi, and Knutson 2017). Therefore, reduction in hunched posture by WS may also be through downregulation of eotaxin as revealed in our study.

The signs of peripheral neuropathy such as numbness and weakness in mice were observed by abnormal behavior and lack of relocation on PTX-administration (Cavaletti et al., 1995; Höke 2012). The peripheral neuropathy is reported to be associated with MCP-1 induction (Zhang et al., 2013), IL-1β release (Hangping et al., 2020; Starobova et al., 2021), and high levels of IL-6 (Zheng et al., 2021). Both the extracts of WS significantly reduced the signs of peripheral neuropathy accompanied by reduction in PTX-induced high levels of MCP-1, IL-1β, and IL-6. Furthermore, KC was found to have a central role in the pathophysiological process of peripheral neuropathy also evident by a reduction in neuropathic pain upon pharmacological blockade of KC receptor- CXCR2 by the selective antagonist (Manjavachi et al., 2014; Piotrowska et al., 2019). ARA significantly reduced the increased levels of KC thereby signs of peripheral neuropathy induced by PTX. The prevention of chemotherapy-induced peripheral neuropathy is correlated with the active IL-4/STAT6 signaling pathway (Shi et al., 2018). Therefore, the reduced sign of peripheral neuropathy in mice on administration of ARH was evident by significant increase in plasma levels of IL-4. In addition, the chemokine RANTES is directly associated with the neuropathic pain (Liou et al., 2013). Though PTX was unable to induce RANTES in mice, WSA and WSH significantly reduced the RANTES expression. Therefore, the role of WS in reducing the neuropathic pain by RANTES depletion is worth investigating.

Chemotherapy-induced alopecia remains a major concern in cancer therapeutics (West 2017). IL-1α contributes to inflammatory alopecia (hair loss) by inhibiting the proliferation of hair follicles (Harmon and Nevins 1993). High serum levels of IL-4, IL-5, and IL-6 are also reported in patients with alopecia (Ito et al., 2020). AR significantly decreased IL-1α whereas WS significantly decreased IL-1α, IL-4, IL-5, and IL-6 in mouse plasma. This implies the possible function of aforementioned cytokines in preventing PTX-induced alopecia by AR and WS.

### Implications in Clinical Management of Cancer

The *Rasayana* effect implies the rejuvenating and adaptogenic property (Saggam et al., 2021). It is an ability to achieve physiological homeostasis to restore health (Rege et al., 1999). The *Rasayana* effects of AR and WS are evident by exhibiting rejuvenating and adaptogenic potential endorsed by Ayurveda (Chulet and Pradhan 2009). The present study helped to generate scientific evidence of the *Rasayana* concept that emphasizes on strengthening physiological homeostasis by preventing myelosuppression and morbidity (Balasubramani et al., 2011). It also highlighted the importance of Ayurveda principles and recommended dosage regimen to achieve desired effects.

A couple of studies depicted the antitumor activities of AR with or without clinically utilised chemotherapeutic drugs (Mitra et al., 2012; Benil et al., 2020). Similarly, our team has previously mapped several pharmacological activities of WS reported in various cancer models indicating its ability to influence the classical hallmarks of cancer (Saggam et al., 2020). The clinical benefits of WS have been reported wherein marked improvement in quality of life was seen in cancer patients undergoing PTX-based chemotherapy regimen (Biswal et al., 2013). These results suggest a potential role of an Integrative Oncology approach using Ayurveda-based therapeutic adjuvants in mitigating the adverse effects of chemotherapy (Winters 2006; Saggam et al., 2020). However, the mechanism(s) underlying these clinical observations have not been well-studied.

The preclinical mouse model employed in our study has been utilised for investigating the pathophysiology of PTX-induced myelosuppression (Ray et al., 2011). Several studies have indicated that this experimental model recapitulates the clinical scenarios in this with reference to biochemical parameters, immuno-regulation, cytokine levels, inflammation and overall behaviour (Burke and Balkwill 1996; Frese and Tuveson 2007; Höke 2012; Lampreht Tratar, Horvat, and Cemazar 2018; Ireson et al., 2019).

Previously published clinical reports on PTX-induced changes in myelosuppressive immune response allow us to intuitively extrapolate our preclinical results into clinical translation. For example, patients with cyclic neutropenia showed elevated levels of G-CSF in neutropenic phase (Watari et al., 1989). In addition, breast cancer patients undergoing PTX treatment have been reported to demonstrate increased levels of GM-CSF in blood (Tsavaris et al., 2002). Indeed, the results of our preclinical study in mice are in alignment with these reported clinical scenarios. In this study, we have observed that the AR and WS extracts prevented neutropenia by normalizing the levels of colony stimulating factors (namely G-CSF and GM-CSF) which were elevated by PTX. Therefore, it is plausible to postulate that co-administration of AR and WS extracts with colony-stimulating factors may have salutary effects in managing chemotherapy-induced myelosuppression. Furthermore, carefully designed molecular studies will shed light on the mechanism(s) underlying the observed protective effect of AR and WS extracts in PTX-induced myelosuppression.

In addition, we observed alterations in cytokine levels associated with immune response which are in alignment with previously reported clinical literature. Marked increase in IL-6 and IL-10 levels subsequent to PTX administration in cancer patients have been reported to be associated with joint pain and fatigue (Tsavaris et al., 2002; Pusztai et al., 2004). In our study, WS extract was found to mitigate PTX-induced elevation of IL-6 and IL-10 levels and restored normal posture (reduced stiffness that occurred due to joint pain) and activity (reduced tiredness that occurred due to fatigue) in mice. In concurrence with the clinical reports from cancer patients, we similarly observed an increase in IL-10 levels after PTX dosing (Pusztai et al., 2004). Furthermore, we observed that the PTX-induced elevation in IL-10 level was not observed when mice were dosed with WS extract prior to or during the course of PTX regimen.

Our study indicates that AR and WS extract offer protective effects against PTX-induced myelosuppression as indicated by experimental observations related to total leukocytes, neutrophils, cytokines and morbidity in mice. These promising preclinical observations potentially lay the foundation for well-designed clinical studies to investigate beneficial effects of Ayurveda-based *Rasayana* botanicals in cancer patients undergoing PTX-associated chemotherapy regimens. Such clinical studies along with molecular pharmacology investigations will be needed in future for exploring the promise of Ayurveda-based botanicals as therapeutic adjuvants in cancer management.

## Conclusion

In conclusion, the present study revealed that AR and WS markedly increased the tolerance of mice towards PTX administration. The clinical adverse effects of PTX such as myelosuppression, fatigue (extreme tiredness), hair loss, peripheral neuropathy (numbness and weakness), pain in muscles and joints etc. can be managed by pre-administration of AR and WS (Markman 2003). Thus, AR and WS are promising cancer adjuvants to enhance the efficacy and reduce the adverse effects of chemotherapy. Further studies on the effects of AR and WS on PTX-induced myelosuppression in a cancer model are needed. Our study suggests well-designed clinical trials with a focus on molecular mechanisms as a future step towards safer and more effective cancer management.

## Data Availability

The original contributions presented in the study are included in the article/Supplementary Material, further inquiries can be directed to the corresponding author.

## Supplementary Material

Supplementary Data Sheet 1 The scoring system of each parameter.

Supplementary Data Sheet 2 The morbidity score (0–3) of each parameter/animal/group/day.

Supplementary Data Sheet 3 The summation of score of all five parameters depicted as total morbidity score of each animal/group/day.

Supplementary Data Sheet 4 The mean and standard deviation of total morbidity score of each group/day.
